# The Proteoglycans Biglycan and Decorin Protect Cardiac Cells against Irradiation-Induced Cell Death by Inhibiting Apoptosis

**DOI:** 10.3390/cells13100883

**Published:** 2024-05-20

**Authors:** Renáta Gáspár, Petra Diószegi, Dóra Nógrádi-Halmi, Barbara Erdélyi-Furka, Zoltán Varga, Zsuzsanna Kahán, Tamás Csont

**Affiliations:** 1 Department of Biochemistry, Albert Szent-Györgyi Medical School, University of Szeged, H-6720 Szeged, Hungary; gaspar.renata@med.u-szeged.hu (R.G.); dioszegi.petra@gmail.com (P.D.); halmi.dora@med.u-szeged.hu (D.N.-H.); erdelyi.furka.barbara@med.u-szeged.hu (B.E.-F.); 2 Interdisciplinary Centre of Excellence, University of Szeged, H-6720 Szeged, Hungary; 3 Department of Oncotherapy, University of Szeged, H-6720 Szeged, Hungary; varga.zoltan@med.u-szeged.hu (Z.V.); kahan.zsuzsanna@med.u-szeged.hu (Z.K.)

**Keywords:** proteoglycan, biglycan, decorin, radiation-induced heart disease, cardioprotection, apoptosis

## Abstract

Radiation-induced heart disease (RIHD), a common side effect of chest irradiation, is a primary cause of mortality among patients surviving thoracic cancer. Thus, the development of novel, clinically applicable cardioprotective agents which can alleviate the harmful effects of irradiation on the heart is of great importance in the field of experimental oncocardiology. Biglycan and decorin are structurally related small leucine-rich proteoglycans which have been reported to exert cardioprotective properties in certain cardiovascular pathologies. Therefore, in the present study we aimed to examine if biglycan or decorin can reduce radiation-induced damage of cardiomyocytes. A single dose of 10 Gray irradiation was applied to induce radiation-induced cell damage in H9c2 cardiomyoblasts, followed by treatment with either biglycan or decorin at various concentrations. Measurement of cell viability revealed that both proteoglycans improved the survival of cardiac cells post-irradiation. The cardiocytoprotective effect of both biglycan and decorin involved the alleviation of radiation-induced proapoptotic mechanisms by retaining the progression of apoptotic membrane blebbing and lowering the number of apoptotic cell nuclei and DNA double-strand breaks. Our findings provide evidence that these natural proteoglycans may exert protection against radiation-induced damage of cardiac cells.

## 1. Introduction

Cancer is a global public health issue, being among the leading causes of death worldwide [1]. Breast and lung cancers are malignant thoracic tumors with constantly increasing incidence and are the primary causes of cancer-related mortality globally [2,3]. Although several advanced treatment strategies (e.g., targeted therapy and immune-checkpoint inhibition therapy) have become available over the past years, conventional modalities (i.e., chemotherapy, radiotherapy, surgical resection, or their combination) still play a fundamental role in the therapy of such malignancies [4]. Irradiation is commonly used as a therapeutic measure in the multidisciplinary treatment of thoracic tumors [5]. Despite the technological development aiming to reduce the harmful effects of irradiation on neighboring healthy tissues, patients who undergo radiation therapy are still at risk of such undesirable tissue damage. In the case of chest irradiation, the most severe presentation of these adverse effects is radiation-induced heart disease (RIHD), which may manifest in a variety of cardiac conditions, such as cardiomyopathy, conduction abnormalities, and valvular diseases [6]. The pathomechanism of RIHD relies on the excess production of reactive oxygen and nitrogen species causing oxidative/nitrative stress, that can culminate in inflammation and damage the DNA as well as intracellular organelles, eventually leading to cell death, often via apoptosis [7,8]. Ionizing radiation has been reported to affect the extracellular matrix (ECM) of the cardiac tissue as well, contributing to cardiac fibrosis [9]. Based on these findings, several potential cardioprotective agents (e.g., statins, angiotensin-converting enzyme inhibitors, dexrazoxane, and antioxidants) have been examined in RIHD [10,11,12]. In spite of these research efforts, so far there is no clinically applicable drug available to attenuate the harmful effects of irradiation on the heart [13].

The ECM is a dynamic, complex network of multidomain macromolecules forming a structurally stable meshwork that provides mechanical support for tissues. In addition, it functions as a reservoir of bioactive compounds, which control the composition of the matrix as well as a variety of cellular functions including cell proliferation, differentiation, and cell death [14]. Small leucin-rich proteoglycans (SLRPs) are extensively investigated ECM components [15], which affect various cellular mechanisms via interaction with numerous proteins (e.g., collagen, transforming growth factor beta (TGF-β), and receptor tyrosine kinases) [16], promote ECM assembly, maintain its integrity, and participate in tissue remodeling after pathophysiological processes [16]. Biglycan (BGN) and decorin (DCN) are the most investigated members of class I SLRPs, with remarkably similar leucine-rich core proteins (i.e., with ≈57% homology) [17,18]. In BGN, two glycosaminoglycan chains are attached to the core protein, while in DCN, only one is attached. BGN has been shown to regulate inflammatory processes, vascular smooth muscle growth and migration, bone mineralization, as well as muscle development and regeneration [19,20,21]. In addition, DCN has been demonstrated to modulate inflammation, fibrosis, and proliferation; therefore, it is thought to exert anti-tumorigenic effects [22,23]. We and others have demonstrated previously that BGN and DCN show cardioprotective properties as well. Both SLRPs have been found to exert beneficial effects against ischemia/reperfusion (I/R) injury [24,25,26,27]. Furthermore, BGN has been suggested to be necessary for cardiac remodeling after myocardial infarction [28]. In addition, BGN has been shown to enhance the expression of several cardioprotective genes and proteins including nitric oxide synthases and the anti-apoptotic members of the Bcl-2 family [29].

Since there are overlapping molecular events in the pathogenesis of ischemia/reperfusion injury and RIHD, both BGN and DCN seem to be promising candidates to beneficially affect RIHD. Therefore, here we examined whether BGN and DCN can protect cardiac cells against irradiation-induced damage. As apoptosis is thought to play a key role in radiation-induced cardiomyocyte loss, we also aimed to investigate if the tested proteoglycans modulate the progression of programmed cell death.

## 2. Materials and Methods

### 2.1. Cell Culture

H9c2 rat cardiomyoblasts (ATCC, Sigma-Aldrich, St. Louis, MO, USA, Cat#CRL-1446) were cultured in Dulbecco’s modified Eagle’s medium (DMEM; Lonza, Basel, Switzerland) supplemented with 10% *v/v* fetal bovine serum (FBS; EuroClone, Pero, Italy, ECS0180L), 1% *v/v* antibiotic/antimycotic (Sigma-Aldrich, St. Louis, MO, USA, A5955), and 200 nM *L*-glutamine solution (Sigma-Aldrich, St. Louis, MO, USA, G7513). Cells were cultured in 25 cm^2^ and 75 cm^2^ tissue culture flasks (Techno Plastic Product, Trasadingen, Switzerland) until they reached 70–80% confluence. Cells from passage P15–18 were seeded at a density of 4 × 10^3^ cells/well in 96-well plates for viability measurement and 5 × 10^4^ cells/well in 6-well plates for a membrane blebbing assay. Cells were also seeded into 24-well plates containing coverslips at a density of 2 × 10^4^ cells/well for the assessment of their nuclear morphology and immunocytochemistry.

### 2.2. In Vitro Model of Radiation-Induced Heart Disease

Two days after cell seeding, cardiomyoblasts were exposed to a single dose of 10 Gray (Gy) irradiation with a dose rate of 6 Gy per minute using a linear accelerator (Varian Clinac DHX, Varian Medical Systems, Palo Alto, CA, USA) [30]. Plates were irradiated with 6 MV energy photon beams using opposing field technique. To achieve a homogeneous dose distribution, 2 cm thick polymethylmethacrylate (PMMA) sheets were applied above and below the sample. After the irradiation, cells were treated with 0.001–0.1 nM BGN (Sigma-Aldrich, St. Louis, MO, USA, Cat#B8041) or DCN (Sigma-Aldrich, St. Louis, MO, USA, Cat#D8428) based on previously published plasma concentrations [31,32,33,34] and preliminary experiments or their vehicle solution. To determine the effect of irradiation, a group of cells was kept under control conditions and treated with the vehicle of BGN (distilled water) or DCN (phosphate buffered saline; PBS). The treatment was maintained for 48 h and then a viability assay was performed to investigate the potential cytoprotective effects of BGN and DCN against irradiation-induced cell death. Furthermore, to investigate the potential cytoprotective effects of BGN and DCN against irradiation-induced apoptotic cell death, the membrane blebbing assay, phosphorylated histone 2A variant X (γ-H_2_AX) immunostaining and 4′-6-diamidino-2-phenylindole (DAPI) staining, as well as Western blot were performed.

### 2.3. Viability Assay

To investigate cell viability, 3-(4,5-dimethylthiazol-2-yl)-2,5-diphenyltetrazolium bromide (MTT; Sigma-Aldrich, St. Louis, MO, USA, Cat#M2128) assay was used. Following irradiation and treatment of the cells with either BGN or DCN according to the previously described protocol, the media were replaced by 0.5 mg/mL MTT in 1% *v/v* FBS-containing DMEM for 1 h at 37 °C. The formed formazan crystals were dissolved in dimethyl sulfoxide (Serva, Heidelberg, Germany, Cat#67-68-5), and the absorbance was determined using a plate reader (BMG ClarioStar Plus, BMG Labtech, Ortenberg, Germany). Data were normalized to the average viability detected in the non-irradiated, vehicle-treated control group. Viability in each experimental group was expressed as percentage of the non-irradiated control group.

### 2.4. Investigation of Membrane Blebbing

To determine the potential effect of SLRPs on irradiation-induced apoptotic cell death, characteristic apoptosis-induced morphological alterations termed membrane blebbing were examined. Cardiac cells were exposed to 10 Gy irradiation followed by treatment with 0.001 nM BGN or 0.003 nM DCN or their vehicle. To determine the effect of irradiation, a group of cells was kept under control conditions and treated with the vehicle of BGN/DCN. At the end of the protocol, cells were washed with warm PBS twice and were collected from 6-well plates using 0.25% trypsin-EDTA solution (Corning, Corning, NY, USA, cat#25-053-Cl) and centrifuged for 5 min at room temperature (RT) (400× *g*). After the removal of supernatants, cell pellets were resuspended in growing media. To visualize apoptotic membrane changes, the resuspended cells were transferred to slides and 5–8 fields were captured using a Leica DMi1 inverted light microscope (Leica Mycrosystems, Wetzlar, Germany). An observer counted and scored the cells in a blinded manner according to characteristic membrane and cell morphological changes using the Image J Software 1.53e with a cell counter plugin (National Institutes of Health, Bethesda, MD, USA). The cells were scored based on the 5 stages of membrane blebbing [35,36,37]. The first two stages involved rounded cells with intact, sharp cell contours (Stage 1—healthy), as well as those which had unchanged shape and size, but showed slight alterations in their membranes’ refraction (Stage 2—altered membrane surface). The cells grouped into the third category showed peripheral circular bulges (Stage 3—cell surface blebbing) and were considered to be reversibly damaged. However, cells showing dynamic blebs indicative of the irreversible, late apoptotic phase were sorted into the fourth group (Stage 4—dynamic blebbing). Cells showing drastically changed shape or signs of final fragmentation were classified into the fifth category (Stage 5—final fragmentation). The number of cells in each stage was expressed as a percentage of the total cell count.

### 2.5. γ-H2AX Immunocytochemistry and Assessment of Apoptotic Nuclear Morphology

For immunocytochemical detection of irradiation-induced DNA double-strand breaks and morphological changes in cell nuclei, the cells were seeded onto glass coverslips at a density of 2 × 10^4^ cells/well and grown in 24-well plates. The cells were exposed to 10 Gy irradiation and treated with 0.001 nM BGN, 0.003 nM DCN, or their vehicle. To determine the effect of irradiation, a group of cells was kept under stress-free conditions and was treated with the vehicle of BGN/DCN. At the end of the protocol, cells on coverslips were fixed using 4% paraformaldehyde (Alfa Aesar, Haverhill, MA, USA, Cat#30525-89-4) (20 min, RT). Immunofluorescence staining of γ-H2AX was used to determine the ratio of cells showing DNA double-strand breaks, while the irradiation-induced morphological changes in the cell nuclei were examined by DAPI staining. For immunocytochemistry, cells were permeabilized with 0.3% Triton X-100—PBS (20 min, RT). Coverslips were washed with PBS three times and blocked in 5% bovine serum albumin-containing PBS (BSA; VWR, Radnor, PA, USA, Cat#9048-46-8) for 30 min at RT. The cells were incubated with γ-H2AX primary antibody (Thermo Fisher Scientific, Waltham, MA, USA, Cat#MA5-33062) in a humidified chamber (1:300, overnight, 4 °C). The cells then were washed with PBS, followed by incubation with Alexa 488 Fluorophore-Conjugated Goat Anti-Rabbit secondary antibody (Cell Signaling Technology, Danvers, MA, USA, Cat# 4412) (1:600, 40 min, RT). The cell nuclei were stained with DAPI solution (Abcam, Cambridge, UK, Cat#ab228549) (1:10,000, 10 min, RT). After three further washing steps, coverslips were covered with Mounting Medium and samples were visualized using NIKON Eclipse Ti-E microscope (Nikon Instruments Inc., Tokyo, Japan). A total of 5–8 fields per slide were captured using the same exposition time for all the samples. Pictures were analyzed using Image J software with a cell counter plugin (National Institutes of Health, Bethesda, MD, USA). To assess nuclear morphology, the number of apoptotic nuclei was determined and normalized to total cell count. Cell nuclei exhibiting either nuclear shrinkage, DNA fragmentation or apoptotic body formation were categorized as ones showing apoptotic nuclear morphology [38]. During the analysis of DNA double-strand breaks, the number of γ-H2AX positive nuclei was determined and expressed as percentage of total cell count [39,40].

### 2.6. Western Blotting

H9c2 cardiomyoblasts were grown in 75 cm^2^ tissue culture flasks and subjected to 10 Gy irradiation, followed by 48 h treatment with BGN/DCN or their vehicle. Preparation of Western blot samples and protein concentration measurement using a BCA Protein Assay Kit (Thermo Fisher Scientific, Waltham, MA, USA, Cat#23225) was performed as described previously [25]. The proteins were separated in 10% polyacrylamide gels, followed by blotting onto 0.45 µm pore-size polyvinylidene fluoride membranes (35 V, 60 min, RT). Membranes were then cut horizontally according to molecular weights of the target proteins. Incubation in 0.01% Tween20-containing TBS supplemented with 5% *v/v* BSA (60 min, RT) was used to block non-specific binding. After washing, the membranes were incubated with specific primary antibodies against Bcl-2 associated X (BAX, Cell Signaling Technology, Danvers, MA, USA, Cat#14796), GAPDH (Cell Signaling Technology, Danvers, MA, USA, Cat#2118) (1:10,000, 16 h, 4 °C), and tubulin (Cell Signaling Technology, Danvers, MA, USA, Cat#2144) (1:2000, 16 h, 4 °C) dissolved in 1% *v/v* BSA. Incubation with Horseradish Peroxidase (HRP)-conjugated Goat anti-Rabbit secondary antibody (DAKO, Glostrup, Denmark, Cat#P0046; 120 min, RT) was carried out, followed by incubation with LumiGlo 20X reagent to enhance the chemiluminescent reaction. Finally, the membranes were exposed to X-ray films for detection. All the films were scanned (400 dpi). Quantity One software 4.4.0.36 (Bio-Rad Company, Hercules, CA, USA) was used to measure the density of each protein band. The measured density was normalized to the expression of the corresponding housekeeping controls. BAX protein levels in the irradiated groups were presented as a percentage of the average expression observed in the control groups. Western blots were carried out using 4 biological replicates harvested from 2 independent experiments. Two technical replicates were performed in each case.

### 2.7. Statistical Analysis

All data are expressed as mean + standard error of the mean (S.E.M.). A statistical analysis was performed with GraphPad Prism 8 (GraphPad Software, GraphPad Software Ltd., La Jolla, CA, USA). The data distribution was examined using the Shapiro–Wilk normality test. Comparisons involving two groups were analyzed using an unpaired *t* test (normal distribution). Comparisons involving more than two groups were analyzed using one-way ANOVA with Dunnet’s post hoc test (normal distribution) or the Kruskal–Wallis test with Dunn’s post hoc test (non-normal distribution). *p* < 0.05 was considered to be significant.

## 3. Results

### 3.1. Both BGN and DCN Attenuate Radiation-Induced Cell Death Dose-Dependently

**Figure 1:**
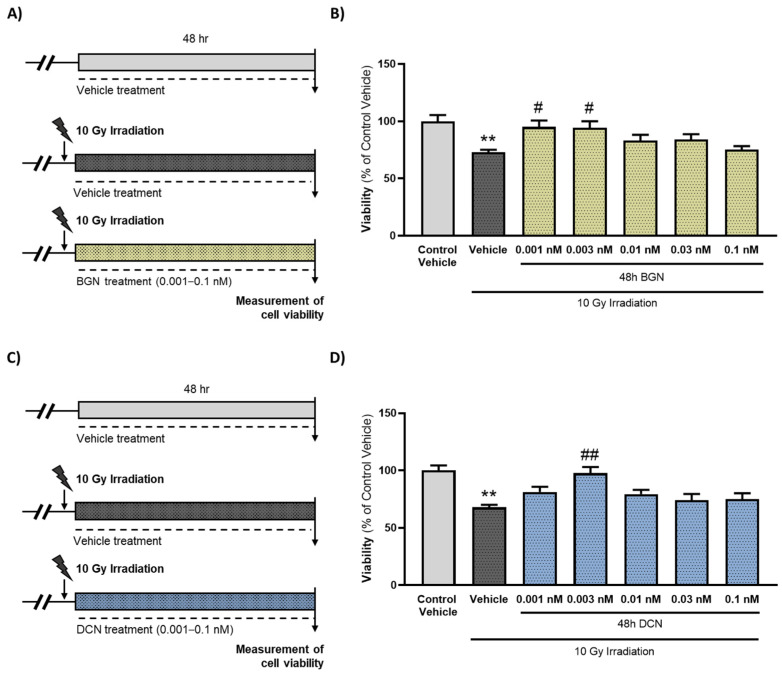
Treatment with either BGN or DCN attenuates irradiation-induced death of cardiomyoblasts. (**A**,**C**) H9c2 cardiomyoblasts were exposed to a single dose of 10 Gy irradiation, followed by a 48 h long treatment with SLRPs or their vehicle. Cells of the non-irradiated control group were kept under stress-free conditions and received vehicle treatment accordingly. Both (**B**) BGN and (**D**) DCN treatments were shown to provide cardiocytoprotective effects at different concentrations. Data were expressed as mean + S.E.M. and compared to non-irradiated or irradiated vehicle-treated groups as appropriate; ** *p* < 0.01 vs. non-irradiated vehicle, # *p* < 0.05 vs. 10 Gy + vehicle, ## *p* < 0.01 vs. 10 Gy + vehicle (BGN treatment: *n* = 33–49 from 5 separated experiments, DCN treatment: *n* = 37–63 from 6 separated experiments; Kruskal–Wallis Test, Dunn’s multiple comparisons test).

The irradiation of cardiac cells caused an approximately 25–30% decrease in viability. Both the SLRPs were shown to exert a dose-dependent effect on the viability of the cardiac cells subjected to irradiation. The most protective doses against radiation-induced cell death were 0.001 nM in the case of BGN (Figure 1B) and 0.003 nM in the case of DCN (Figure 1D) treatment. Higher concentrations (≥0.01 nM) of SLRPs did not affect the viability significantly compared to the corresponding irradiated, vehicle-treated groups.

### 3.2. BGN and DCN Restrain the Radiation-Induced Progression of Apoptotic Membrane Blebbing

**Figure 2:**
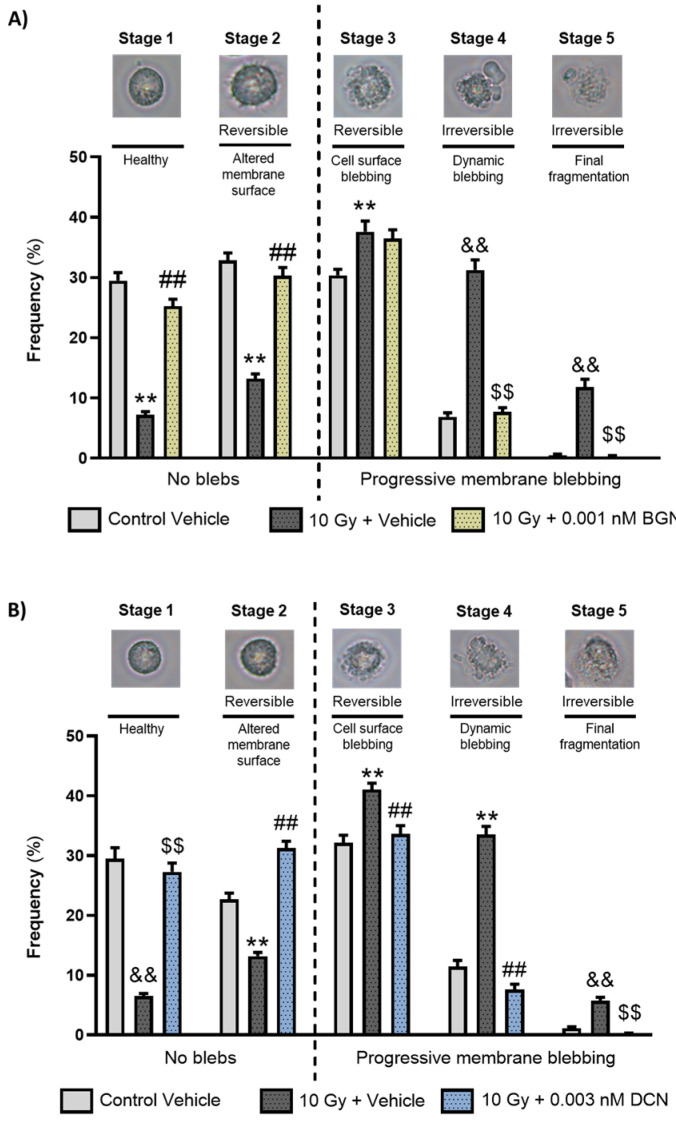
Both biglycan and decorin beneficially affected the irradiation-induced progression of apoptotic membrane blebbing. H9c2 cardiomyoblasts were exposed to a single dose of 10 Gy irradiation, followed by a 48 h long treatment with either 0.001 nM BGN or 0.003 nM DCN, or their vehicle. Cells of the non-irradiated control group were kept under stress-free conditions and received vehicle treatment accordingly. Both (**A**) BGN and (**B**) DCN treatments seemed to induce a substantial decrease in the irradiation-induced progression of membrane blebbing, especially in Stages 4 and 5 that are associated with irreversible damage. Number of cells in each stage of membrane blebbing was expressed as percentage of total cell number. Data were expressed as mean + S.E.M and compared to non-irradiated or irradiated vehicle-treated groups as appropriate; ** *p* < 0.01 vs. non-irradiated Vehicle, ## *p* < 0.01 vs. 10 Gy + Vehicle, one-way ANOVA, Dunnett’s multiple comparisons test; && *p* < 0.01 vs. non-irradiated Vehicle, $$ *p* < 0.01 vs. 10 Gy + Vehicle, Kruskal–Wallis Test, Dunn’s multiple comparisons test (*n* = 9 from 3 separated experiments, 5–8 view field/sample).

As both BGN and DCN were shown to protect cardiomyoblasts against radiation-induced cell death, we aimed to examine whether any of these SLRPs intervene in the process of programmed cell death. First, we assessed their morphological characteristics via the analysis of apoptotic membrane blebbing. A light microscopy investigation performed on living cells revealed that the irradiation of cardiomyoblasts promotes the progression of membrane blebbing (Figure 2A,B), significantly decreasing the ratio of morphologically intact cells and increasing the number of cardiomyoblasts undergoing progressive membrane blebbing. BGN treatment at a concentration of 0.001 nM was shown to reduce the harmful effects of irradiation significantly (Figure 2A). A similar protective effect was observed in the case of 0.003 nM DCN treatment (Figure 2B), causing a substantial decrease in the radiation-induced progression of membrane blebbing. The protective effect was the most prominent in case of the irreversibly damaged cell population (i.e., stages 4 and 5).

### 3.3. BGN and DCN Attenuate Radiation-Induced Apoptotic Alterations in Nuclear Morphology

**Figure 3:**
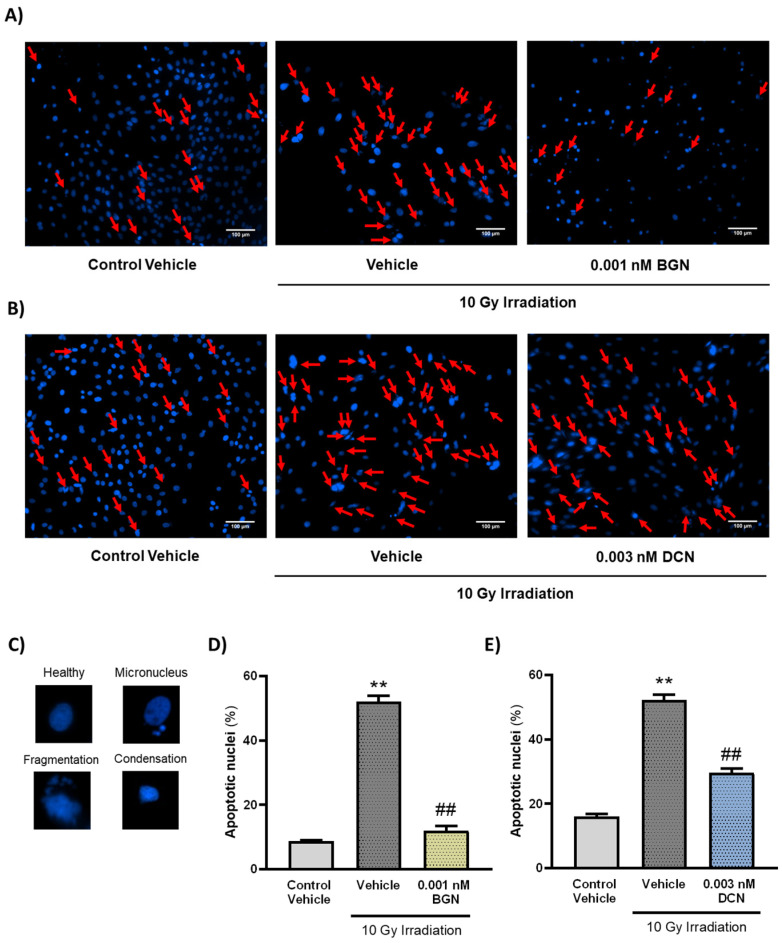
Both BGN and DCN diminished the irradiation-induced alterations in nuclear morphology. H9c2 cardiomyoblasts were exposed to a single dose of 10 Gy irradiation, followed by a 48 h long treatment with SLRPs or their vehicle. Cells of the non-irradiated control group were kept under stress-free conditions and received vehicle treatment accordingly. (**A**–**C**) Representative pictures demonstrate the frequency of damaged nuclei, as well as reference images of nuclei showing apoptotic morphology. Both (**A**,**D**) BGN and (**B**,**E**) DCN treatments were shown to reduce the irradiation-induced increase in the ratio of cell nuclei showing apoptotic morphology. Scale: 100 µm. Red arrows indicate nuclei showing apoptotic morphology. Data were expressed as mean + S.E.M and compared to non-irradiated or irradiated vehicle-treated groups as appropriate; ** *p* < 0.01 vs. non-irradiated Vehicle, ## *p* < 0.01 vs. 10 Gy + Vehicle (*n* = 4 from 2 separated experiments, 5–8 view field/sample, Kruskal–Wallis Test, Dunn’s multiple comparisons test).

To further investigate the effects of the tested SLRPs on apoptosis, their nuclear morphology was assessed. Nuclei were analyzed after visualization using DAPI staining and categorized upon whether they show morphological hallmarks of apoptosis (i.e., micronuclei formation and DNA condensation or fragmentation). A single dose of 10 Gy irradiation was found to increase the number of nuclei showing apoptotic morphology considerably (Figure 3A–E). Both 0.001 nM BGN (Figure 3A,D) and 0.003 nM DCN (Figure 3B,E) treatments were shown to diminish the effects of irradiation on apoptotic nuclear morphology, substantially reducing the ratio of apoptotic nuclei.

### 3.4. Both BGN and DCN Attenuate the Radiation-Induced Increase in the Number of DNA Double-Strand Breaks

**Figure 4:**
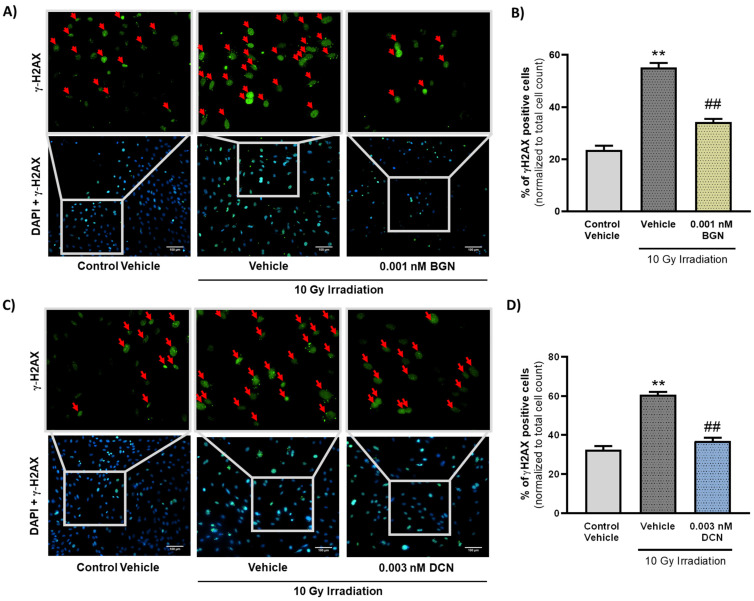
SLRPs decrease the irradiation-induced elevation in the frequency of DNA double-strand breaks. H9c2 cardiomyoblasts were exposed to a single dose of 10 Gy irradiation, followed by a 48 h long treatment with SLRPs or their vehicle. Cells of the non-irradiated control group were kept under stress-free conditions and received vehicle treatment accordingly. Both (**A**,**B**) BGN and (**C**,**D**) DCN treatments were found to decrease the irradiation-induced increase in the ratio of cells showing γ-H2AX positivity (indicated with red arrows). Scale: 100 µm. Data were expressed as mean + S.E.M and compared to non-irradiated or irradiated vehicle-treated groups as appropriate; ** *p* < 0.01 vs. non-irradiated Vehicle, ## *p* < 0.01 vs. 10 Gy + Vehicle (*n* = 4 from 2 separated experiments, 5–10 view field/sample, Kruskal–Wallis Test, Dunn’s multiple comparisons test).

Critical damage of the DNA is considered as a typical apoptosis-triggering lesion [41]. Therefore, we assessed the frequency of DNA double-strand breaks in our different experimental groups to examine whether SLRPs influence the induction of programmed cell death or, rather, impact its progression exclusively. Immunostaining of γ-H2AX revealed that irradiation induces a substantial increase in the ratio of γ-H2AX positive nuclei containing DNA double-strand breaks (Figure 4A–D). The ratio of γ-H2AX positive cells was shown to decrease significantly in the 0.001 nM BGN-treated group of cells compared to their vehicle-treated irradiated controls (Figure 4A,B). DCN treatment at a concentration of 0.003 nM was found to exert similar beneficial effects, substantially reducing the number of cell nuclei showing DNA double-strand breaks (Figure 4C,D).

### 3.5. BGN and DCN Treatments Reduce the Radiation-Induced Increase in Proapoptotic BAX Levels

**Figure 5:**
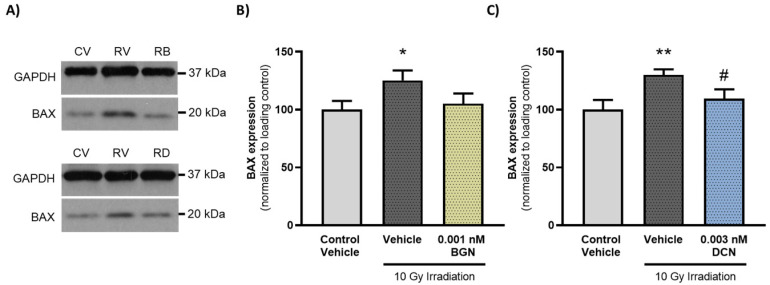
BGN and DCN treatments reduce the irradiation-induced elevation in proapoptotic BAX levels. H9c2 cardiomyoblasts were exposed to a single dose of 10 Gy irradiation, followed by a 48-h long treatment with SLRPs or their vehicle. Cells of the non-irradiated control group were kept under stress-free conditions and received vehicle treatment accordingly. (**A**) Representative pictures (CV: control vehicle, RV: irradiated vehicle-treated, RB: irradiated BGN-treated, and RD: irradiated DCN-treated groups). Both (**A**,**B**) BGN and (**A**,**C**) DCN treatments were found to substantially reduce the irradiation-induced increase in the expression of proapoptotic BAX. Protein expression was presented as percentage of BAX levels observed in the non-irradiated control groups. Data were expressed as mean + S.E.M and compared to non-irradiated or irradiated vehicle-treated groups as appropriate; * *p* < 0.05 vs. non-irradiated Vehicle, ** *p* < 0.01 vs. non-irradiated Vehicle, # *p* < 0.05 vs. 10 Gy irradiation + Vehicle (*n* = 4 from 2 separated experiments, unpaired *t* test).

To further support that administration of either BGN or DCN counteracts the irradiation-induced aggravation of apoptosis, we determined the protein expression of a key proapoptotic mediator, BAX, which has been shown to mediate radiation-induced apoptosis induction [42]. Investigation of the molecular events revealed that irradiation of cardiac cells increased the expression of proapoptotic BAX significantly (Figure 5A,B). However, 0.001 nM BGN treatment was shown to prevent such an increase in BAX levels, retaining its expression closer to that observed in the control group (Figure 5A). Moreover, 0.003 nM DCN treatment was found to cause a substantial decrease in BAX levels compared to those observed in vehicle-treated irradiated cells (Figure 5B).

## 4. Discussion

In the present study we have demonstrated that both BGN and DCN protect cardiac cells against radiation-induced cellular damage. Although multiple studies have been conducted to analyze the roles of these SLRPs in certain cardiovascular pathologies, including myocardial ischemia and vascular abnormalities, to date, our study is the first to investigate the effect of these proteoglycans on radiation-induced cardiac cell damage.

Our results imply that exogenous administration of either BGN or DCN dose dependently increases the survival of cardiac cells exposed to irradiation. This suggests that the application of SLRPs may exert protection against cardiac damage, which is in line with several other observations proposing BGN- and DCN-derived beneficial effects against various cardiac pathologies, including I/R-induced cardiac damage [24,25,26], type 2 diabetes-induced cardiomyopathy [43], as well as angiotensin II-induced vascular abnormalities and heart failure [44,45]. However, some investigations suggest contradictory SLRP-related cardiovascular effects, e.g., BGN has been reported to contribute to cardiac hypertrophy and fibrosis, as well as atherosclerosis [46,47]. Nonetheless, our findings support that SLRPs exert beneficial effects against radiation-induced cardiac abnormalities.

The underlying mechanism of RIHD comprises several intracellular events harmfully affecting cardiomyocytes (e.g., oxidative stress, impairment of the endoplasmic reticulum, mitochondrial dysfunction, subsequent calcium overload, and DNA damage) [48,49]. These collectively promote programmed cell death or apoptosis, eventually leading to the loss of cardiomyocytes [50], thereby contributing to the development of radiation-induced cardiac abnormalities. To test whether the observed BGN- and/or DCN-derived cardiocytoprotection involves the modulation of such radiation-induced proapoptotic events, we assessed the morphological hallmarks of programmed cell death. Our results demonstrated that both BGN and DCN treatment substantially retained the progression of apoptotic membrane blebbing, suggesting that the proposed cardioprotective effect of either examined SLRPs might occur at least in part due to antiapoptotic properties. To further confirm this hypothesis, we assessed apoptotic nuclear morphology, which revealed that the administration of either proteoglycan caused a considerable decrease in the ratio of cell nuclei showing signs of apoptosis (i.e., micronuclei formation, condensation, or fragmentation) compared to those observed in irradiated vehicle-treated groups. This antiapoptotic effect is in line with findings demonstrating that overexpression of BGN blocks the sodium nitroprusside-induced increase in nuclear fragmentation and condensation, thereby alleviating NO-induced neuronal apoptotic cell death [51]. As DNA damage is considered to be a driver of apoptosis [41], we performed γ-H2AX staining to reveal if the application of SLRPs affects the radiation-induced increase in DNA double-strand breaks. Our results demonstrated that administration of either BGN or DCN decreased the radiation-induced DNA damage substantially, further supporting their potential antiapoptotic effects in cardiac cells. To confirm our morphology-based findings on the molecular level, we investigated the protein expression of BAX, a key proapoptotic mediator of irradiation-induced apoptosis [42,52], which revealed that both BGN and DCN treatments seem to counteract the irradiation-induced substantial increase in BAX levels. Multiple studies have reported similar SLRP-derived effects on BAX expression in other settings [53,54,55], which in line with our observations suggest that both BGN and DCN suppress apoptotic activity at least in part via the downregulation of key mediators initiating apoptotic cell death.

Overall, our findings are in accordance with other observations suggesting that SLRPs alleviate apoptotic activity. Although limited data are available regarding their protective effect against cardiac apoptosis [25], multiple investigations have been carried out in other settings. For example, nuclear factor kappa B-induced upregulation of BGN was found to reduce the nitric oxide-induced apoptosis of human neuroblastoma cells [56], while DCN has been demonstrated to exert protection against hydrogen peroxide-induced apoptosis of retinal pigment epithelial cells [57]. In addition, administration and increased expression of DCN have been found to limit the degree of apoptosis in tubular epithelial cells of the kidney [58] as well as in endothelial cells [59]. A similar effect was observed by Zhang et al., who reported that DCN treatment attenuates IL-1β-induced apoptosis of rat nucleus pulposus cells [54]. Nonetheless, limited amount of data are available with regards to the thorough mechanism of action through which SLRPs counteract proapoptotic events. Both BGN and DCN have been shown to act on Toll-like receptors (TLR), which have been implicated in their protective effect against cardiac ischemia [25] as well as in their ability to upregulate tumor suppressor genes facilitating DNA repair mechanisms [60]. The effect of SLRPs on apoptosis has been investigated in cancer cells as well, interestingly resulting in contradictory findings. BGN has been reported to exert antiapoptotic effect in HCT116 colon cancer cells [61] and to promote the proliferation of gastric cancer cells [62], suggesting a potential oncogenic role in certain gastrointestinal malignancies. However, opposite effects have been implicated in case of breast cancer, indicating that BGN mediates the antiproliferative effects of embryonic mesenchyme inducing partial breast cancer reversion [63]. In addition, various investigations conducted on cancer cells suggest that DCN does not inhibit but rather promotes apoptosis in the tumor tissue. It has been reported that DCN core protein stimulates apoptosis in human squamous carcinoma cells [22]. In line with this, Yoon et al. published that increased DCN expression stimulates apoptosis in various cancer cell lines (i.e., U343 and U87MG—glioblastoma; A549—lung cancer; Hep3B—liver cancer; and C33A—cervical cancer cells) [64]. These studies indicate that DCN might exert antitumor activity via the modulation of apoptosis adversely in cancer cells. Although it has not been investigated, to date, whether this beneficial effect occurs in the case of thoracic malignancies as well, a potential antitumor effect would further increase the efficacy of future treatments, reducing radiotherapy-induced cardiac damage and preventing cancer progression at the same time.

In summary, our results show that the application of two structurally related SLRPs, BGN and DCN, exert protection against radiation-induced cardiac damage, whose effect seems to involve the attenuation of apoptotic processes. Our results implicate these natural molecules as potential cardioprotective agents suitable for further development.

### Limitations of the Study

We have demonstrated cardiocytoprotective effects of both BGN and DCN against radiation-induced cell damage; however, our work is not without limitations. Further examination of the potential antiapoptotic mechanisms may contribute to clarify the precise sequences of molecular events triggered by SLRPs during radiation-induced apoptosis in cardiac cells. As we presented data obtained from in vitro experiments using H9c2 cells, it would be beneficial to explore in future studies whether SLRPs impact radiation-induced alterations of primary cardiac cells (e.g., neonatal or adult cardiomyocytes) and other cell types (e.g., fibroblasts, endothelial cells, etc.) and to corroborate these findings under in vivo circumstances as well. Despite all the limitations, our results clearly suggest that BGN and DCN protect cardiac cells against radiation-induced cell damage by inhibiting apoptosis.

## 5. Conclusions

In the present study, we have demonstrated that two structurally related SLRPs, BGN and DCN, exert protection against radiation-induced cardiac damage, substantially improving the survival of cardiac cells undergoing irradiation. The observed cardiocytoprotection seemed to involve antiapoptotic signaling, as revealed by the analysis of morphological and molecular hallmarks of programmed cell death. Further investigations are required to analyze whether the examined SLRPs affect other types of cell death and/or survival pathways, to identify their precise molecular mechanism of action, as well as to test their effects in vivo. Nevertheless, according to our present findings, it seems plausible that these natural compounds may be appealing candidates for future development of novel cardioprotective agents.

## Data Availability

Data are available from the corresponding author upon reasonable request.

## Abbreviations

BAX	Bcl-2 associated X
BGN	biglycan
BSA	bovine serum albumin
DAPI	4′-6-diamidino-2-phenylindole
DCN	decorin
ECM	extracellular matrix
FBS	fetal bovine serum
Gy	Gray
I/R	ischemia/reperfusion
MTT	3-(4,5-Dimethylthiazol-2-yl)-2,5-Diphenyltetrazolium Bromide
PBS	phosphate-buffered saline
RIHD	radiation-induced heart disease
RT	room temperature
SLRP	small leucin-rich proteoglycans
TGF-β	transforming growth factor beta
TLR	Toll-like receptor
γ-H2AX	phosphorylated histone 2A variant X
